# Comparative transcriptome analysis of the metal hyperaccumulator *Noccaea caerulescens*

**DOI:** 10.3389/fpls.2014.00213

**Published:** 2014-05-20

**Authors:** Pauliina Halimaa, Daniel Blande, Mark G. M. Aarts, Marjo Tuomainen, Arja Tervahauta, Sirpa Kärenlampi

**Affiliations:** ^1^Department of Biology, University of Eastern FinlandKuopio, Finland; ^2^Laboratory of Genetics, Wageningen UniversityWageningen, Netherlands

**Keywords:** RNA-Seq, deep sequencing, NGS, *Noccaea caerulescens*, *Thlaspi caerulescens*, hyperaccumulation, metal tolerance, Brassicaceae

## Abstract

The metal hyperaccumulator *Noccaea caerulescens* is an established model to study the adaptation of plants to metalliferous soils. Various comparators have been used in these studies. The choice of suitable comparators is important and depends on the hypothesis to be tested and methods to be used. In high-throughput analyses such as microarray, *N. caerulescens* has been compared to non-tolerant, non-accumulator plants like *Arabidopsis thaliana* or *Thlaspi arvense* rather than to the related hypertolerant or hyperaccumulator plants. An underutilized source is *N. caerulescens* populations with considerable variation in their capacity to accumulate and tolerate metals. Whole transcriptome sequencing (RNA-Seq) is revealing interesting variation in their gene expression profiles. Combining physiological characteristics of *N. caerulescens* accessions with their RNA-Seq has a great potential to provide detailed insight into the underlying molecular mechanisms, including entirely new gene products. In this review we will critically consider comparative transcriptome analyses carried out to explore metal hyperaccumulation and hypertolerance of *N. caerulescens*, and demonstrate the potential of RNA-Seq analysis as a tool in evolutionary genomics.

## INTRODUCTION

The Alpine pennycress *Noccaea caerulescens* (previously *Thlaspi caerulescens*) from the Brassicaceae family has been extensively studied at the physiological level for its ability to hyperaccumulate and hypertolerate metals such as Zn, Cd, and Ni (Meerts and Van Isacker, 1997; Lombi et al., 2000; Reeves et al., 2001; Assunção et al., 2003c; Roosens et al., 2003; Escarré et al., 2013). To explore the basis for these traits, a number of plants with differences in hyperaccumulation and hypertolerance have been compared. This includes cross- and intra-species comparisons, and comparisons between accumulators and non-accumulators. The choice of comparators is important and depends on the hypothesis to be tested and methodology used. While deep sequencing techniques have the potential to greatly increase our understanding about the mechanisms of plant metal-related traits and their evolution during the adaptation to different environments, a careful consideration of appropriate comparators becomes increasingly important. A major challenge is to find the genes of interest among those differentially expressed between the plants. In this review we consider the choice of comparators in exploring metal hyperaccumulation and hypertolerance characteristics of *N. caerulescens*, and discuss the pros and cons of the approaches in relation to RNA-Seq analysis. Applying RNA-Seq analysis to *N. caerulescens* populations with contrasting metal tolerance or hyperaccumulation capacities is a superior tool to analyze the evolutionary genomics of plant adaptation strategies to metalliferous soils.

## TRANSCRIPTOME COMPARISON BETWEEN *N. caerulescens* AND NON-TOLERANT NON-ACCUMULATOR PLANTS

The non-accumulator, non-tolerant *Arabidopsis thaliana*, a model plant from the Brassicaceae family, is considered as a good comparator to *N. caerulescens* because of considerable sequence similarity: 87-88% identity in intergenic transcribed spacer regions (Peer et al., 2003), and ca. 88.5% nucleotide identity within transcribed regions (Rigola et al., 2006). Furthermore, genetic resources available for *A. thaliana* are superior compared to any other plant.

One of the first efforts to characterize the *N. caerulescens* transcriptome was made by Rigola et al. (2006), comparing ESTs (expressed sequence tags) of *A. thaliana* and *N. caerulescens* accession La Calamine, a Zn-tolerant Zn hyperaccumulator (Assunção et al., 2003c). Besides two species, the complex comparison thus included Zn hyperaccumulator/non-accumulator pair, Zn tolerant/non-tolerant pair as well as different Zn concentrations. The assembled partial cDNA sequences (unigenes) represented only 13% of the root and shoot transcriptomes, but some interesting findings emerged. Several unigenes showed similarity to genes for which a role in metal hyperaccumulation, tolerance or homeostasis was previously implicated. Three percent of the unigenes corresponded to *A. thaliana* orthologues not known to be expressed, and ca. 8% were considered *N. caerulescens* – specific. An effort was made to estimate transcriptional activity of highly expressed genes from the frequency of their detection but, unlike RNA-Seq, EST analysis provides limited information about transcript abundances.

To identify genes involved in Zn tolerance and/or accumulation, van de Mortel et al. (2006) compared root gene expression profiles of *A. thaliana* and *N. caerulescens* (La Calamine) under Zn excess and deficiency. For both species, a 60-mer oligo DNA microarray covering nearly complete *A. thaliana* transcriptome was used. An uncertainty in this approach was that the probes generally did not hybridize to *N. caerulescens* cDNA as efficiently as to *A. thaliana* cDNA, partly because they were designed to fit less conserved regions of *A. thaliana* transcripts. Furthermore, in the absence of *N. caerulescens* genome data, a marked effort was needed to develop primers to verify the results by PCR, as microarray does not provide accurate information about sequences. Over 2000 genes were significantly differentially expressed between *A. thaliana* and *N. caerulescens*. A challenge remained that most of the differentially expressed genes would not be directly linked to metal homeostasis. Furthermore, considering Zn hyperaccumulation as a constitutive species-level trait in *N. caerulescens* (Rascio and Navari-Izzo, 2011), many metal homeostasis genes being constitutively highly expressed in hyperaccumulators (Hammond et al., 2006; van de Mortel et al., 2006, 2008), Zn-dependence of the expression would not be expected for these genes.

In order to identify genes primarily involved in Cd tolerance, van de Mortel et al. (2008) investigated the root gene expression profiles of *A. thaliana* and *N. caerulescens* (La Calamine) under different Cd and Zn exposures, using the same *A. thaliana* array platform as van de Mortel et al. (2006). The authors concluded that these two species have specific responses to Cd, and emphasized the role of lignin, glutathione and sulfate metabolism. Only 93 out of 409 *N. caerulescens* genes differentially expressed in response to Zn exposures were identical to those reported by van de Mortel et al. (2006), highlighting the fact that differences in plant growth conditions, sampling, but also probe hybridization conditions lead to major differences in the outcomes. Interestingly, although similarities were found in gene expression profiles between *N. caerulescens* and *Arabidopsis halleri*, another metal hyperaccumulator species, compared to *A. thaliana*, there were also clear differences (Weber et al., 2004; Talke et al., 2006), which implied either species-specific differences in the mechanisms of metal hyperaccumulation or methodological differences.

Another non-accumulator plant, *Thlaspi arvense*, has been used as a comparator to *N. caerulescens*. The average similarity between the coding regions of *A. thaliana* and *N. caerulescens* or *T. arvense* genes was calculated by Hammond et al. (2006) as 81.5%. To increase the understanding about Zn hyperaccumulation mechanisms, Hammond et al. (2006) compared shoot transcriptomes of agar- and compost-grown *N. caerulescens* (Viviez population) with *T. arvense* by using an *A. thaliana* array, in which each gene was represented by a set of oligo probes. The array was extensively validated in order to overcome the potential limitations arising from sequence divergence between orthologous genes in *A. thaliana*, *N. caerulescens* and *T. arvense*, as the probe selection was demonstrated to dramatically affect the estimates of differential gene expression. Approximately 5000 differentially expressed genes were found, including several genes previously implicated in Zn homeostasis. Literature comparison indicated that very few of those genes were common to the genes identified when *A. halleri* and *A. thaliana* were compared which, according to Hammond et al. (2006), might have been a consequence of not accounting for sequence divergences between *A. thaliana* and *A. halleri*. Furthermore, although Viviez population shows constitutive Zn hyperaccumulation capacity, it is primarily a Cd hyperaccumulator and thus perhaps not the best comparator to study Zn hyperaccumulation (Schwartz et al., 2003).

Until now, no transcriptome comparisons between *N. caerulescens* and related metal hyperaccumulators are available. In principle, such comparisons might be useful if the plants provide unique accumulation/tolerance profiles. Even with similar profiles, these comparisons might help to reject or support a hypothesis and could tell whether the genetic basis of any specific tolerance or accumulation mechanism is species-specific or more universal. Examples of such plants are the Ni accumulator *N. goesingense* (Lombi et al., 2000) and the Zn/Cd hyperaccumulator *N. praecox*, which accumulates Cd similarly to *N. caerulescens* Ganges accession but is less Cd-tolerant (Vogel-Mikuš et al., 2005). Another comparator of interest is the more distantly related *A. halleri*, which accumulates Cd and Zn but not Ni, is not Ni tolerant, shows different Zn compartmentation, and has differences in the regulation of Cd uptake compared to *N. caerulescens* (Cosio et al., 2004; Marquès et al., 2004; Broadley et al., 2007). Comparing *N. caerulescens* and *A. halleri* at specific tissue- or even single cell level might help determining the specific mechanisms responsible for metal accumulation (e.g., different Zn compartmentation).

## MICROARRAY ANALYSIS VERSUS RNA-SEQ

While cross-species microarray analyses have provided valuable information about *N. caerulescens* and its responses to environment compared to the related non-accumulators *A. thaliana* and *T. arvense*, the approaches have limitations, which may lead to a conclusion that there is a link between metal hyperaccumulation and a specific gene expression when in fact there is not, and vice versa. All microarray-based methods have limitations in terms of sensitivity and dynamic range, but a major challenge in cross-species comparisons is the sequence divergence of orthologous genes and thus the probe design. This can lead to inaccuracies, e.g., in estimating relative transcript abundances and to the lack of identification of novel transcripts.

RNA-Seq analysis is rapidly becoming the method of choice in the cross- and intra-species comparisons. Some advantages are its superior sensitivity and dynamic range. An example of the sensitivity is the *MYB72* gene, which is of interest because the *A. thaliana myb72* loss-of-function mutant exhibits decreased tolerance to Zn and Fe (van de Mortel et al., 2008). No expression of an orthologue could be established in *N. caerulescens* by microarray or low-stringency semi-quantitative RT-PCR (van de Mortel et al., 2008). Low-level expression was, however, found by RNA-Seq analysis, which opens the possibility that this transcription factor is involved in Zn tolerance of *N. caerulescens* (Halimaa et al., 2014). It should be noted that the detection of lowly expressed genes in RNA-Seq analysis is particularly sensitive to sequencing depth and coverage (Rapaport et al., 2013).

**FIGURE 1:**
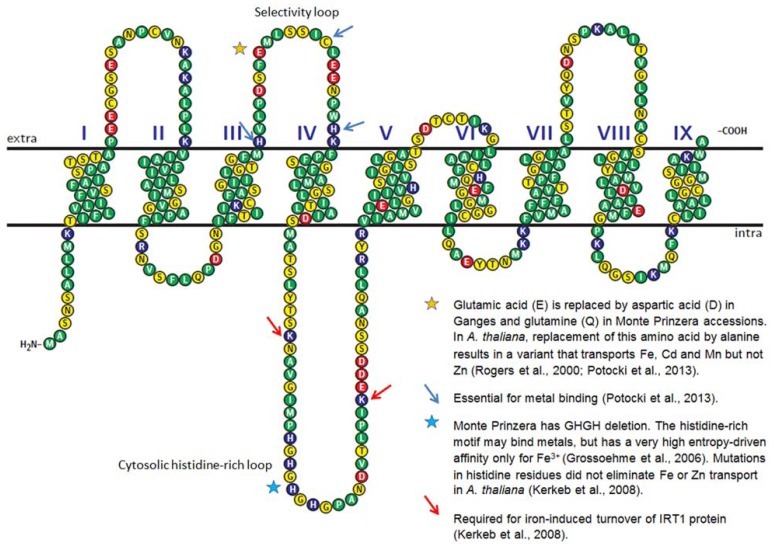
**Predicted secondary structure of *A. thaliana* IRT1 metal transporter**. The amino acid sequence was retrieved from TAIR10 database. The secondary structure of the protein was predicted using HMMTOP 2.0 (Tusnády and Simon, 1998, 2001). The diagram was generated with TEXtopo package (Beitz, 2000). Amino acids known to be important for the function of IRT1 are labeled with stars and arrows. Stars also indicate the locations where *N. caerulescens* accessions differ from *A. thaliana*. The *N. caerulescens* amino acid sequences were derived from Illumina RNA-Seq analysis of three accessions: Lellingen, Ganges, and Monte Prinzera.

Since the output is made available as sequences and read counts rather than signal intensities like in microarrays, RNA-Seq is particularly attractive for species that have not been fully sequenced, such as *N. caerulescens*. Apart from being able to reveal novel transcripts, RNA-Seq can provide detailed information about single nucleotide polymorphisms (SNPs), splice variants and small RNAs. An example of nucleotide polymorphism leading to differences in amino acid sequences is shown in **Figure 1** for IRT1 transcript assembled from Illumina RNA-Seq data in three *N. caerulescens* accessions (Halimaa et al., unpublished). Some of the differences in the corresponding amino acid sequences of this metal transporter might contribute to differences in metal specificity among the accessions, e.g., by influencing the affinity to different metals. In *Arabidopsis*, IRT1 has broad metal specificity (Eide et al., 1996; Korshunova et al., 1999).

The RNA-Seq technology does not fully solve the problems faced with cross-species comparisons. When comparing, e.g., *N. caerulescens* and *A. thaliana* to a common reference genome, the two species will have different mapping properties, complicating the calculation of transcript abundances (Derrien et al., 2012). In cross-species comparisons, the safest approach is thus to compare the profiles of sets of genes rather than transcript levels of individual genes. This problem can be partly overcome by *de novo* assembling the *N. caerulescens* transcriptome. However, this introduces a new challenge of identifying orthologues between the two species, particularly for gene families with many similar sequences (Romero et al., 2012). The reliability of comparisons increases with closer phylogenetic relationship between the plants to be compared (Koch and German, 2013).

## *Noccaea Caerulescens* ACCESSIONS AS THE SOLUTION FOR THE COMPARATIVE RNA-SEQ ANALYSIS

Whereas caution has to be exercised when comparing different species, different *N. caerulescens* accessions and crosses could be almost ideal comparators in RNA-Seq, particularly if the accessions have been self-pollinated for several generations and are physiologically well-characterized. Comparison of *IRT1* sequences showed that, whereas differences were found on average in 12.4% of the nucleotides compared to *A. thaliana*, the polymorphism among three *N. caerulescens* accessions was only 1.7%, apart from a deletion in the histidine motif in Monte Prinzera (**Figure 1**; Halimaa et al., unpublished). Furthermore, when comparing transcript abundances between accessions by mapping the RNA-Seq reads to a common reference genome, the read counts can simply be normalized by the sequencing depth of the library (for a review of normalization methods see Dillies et al., 2012).

**FIGURE 2:**
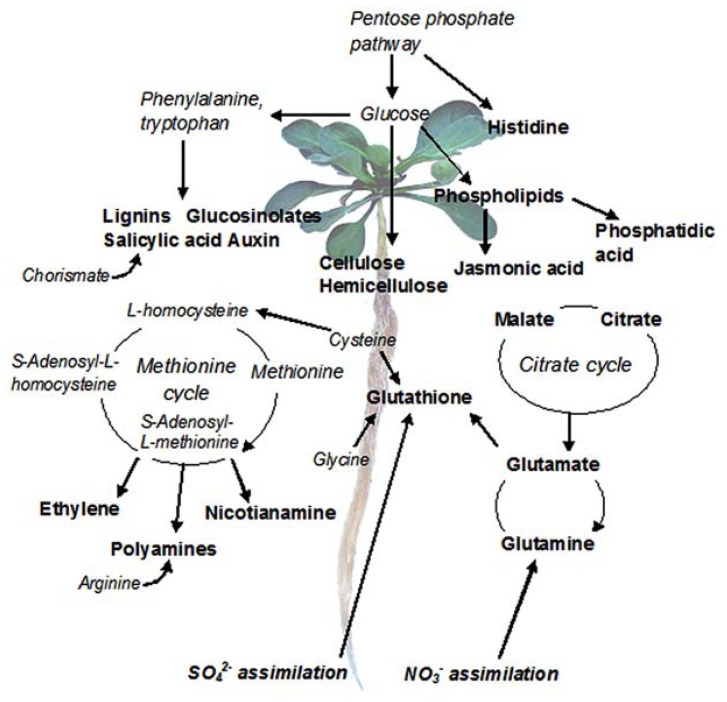
**The most active metabolic pathways in *N. caerulescens* roots**. The data are based on SOLiD RNA-Seq analysis of three biological replicates of three *N. caerulescens* accessions: La Calamine, Ganges, and Monte Prinzera. The read counts were normalized by the size of the libraries and the length of the genes, assuming an equal length for orthologous genes in *A. thaliana* and *N. caerulescens*, and the most highly expressed genes (expression value > 1000) were subjected to a more detailed analysis. The genes were manually linked to metabolic pathways in KEGG (Kyoto Encyclopedia of Genes and Genomes).

Another advantage of RNA-Seq is that different transcripts within one sample can be compared using FPKM normalization, i.e., adjustment by the length of each complete transcript. This can be used, e.g., to answer the question which pathways dominate in a specific tissue. As an example, we have used SOLiD RNA-Seq system to analyze the pathways that dominate in the roots of three *N. caerulescens* accessions (**Figure 2**; Halimaa et al., 2014). The biosynthesis of lignin, glucosinolates, and auxin were among the most highly expressed pathways in all three accessions. Lignin and auxin biosynthesis are related to general root development, while glucosinolate biosynthesis is associated with the Brassicaceae family. Genes related to sulfate and nitrate assimilation were also highly expressed, providing essential raw material to metal ligand synthesis. Nitrate enhances Zn hyperaccumulation in the roots and shoots of *N. caerulescens* (White-Monsant and Tang, 2013). Methionine cycle that generates intermediates to nicotianamine, ethylene, and polyamine synthesis was very highly expressed. Additionally, genes encoding the synthesis of metal ligands (e.g., glutathione, histidine, glutamine, metallothioneins, nicotianamine, and citrate) were abundantly expressed. Furthermore, our data indicated high expression of many genes linked to scavenging of reactive oxygen species (ROS) and related signaling networks. This is in line with previous findings linking salicylate and jasmonate biosynthesis to signaling of glutathione-mediated Ni tolerance (Freeman et al., 2005), and to Cd tolerance (Tolrà et al., 2006), respectively. As all known *N. caerulescens* populations have basal levels of metal tolerance and hyperaccumulation that exceed those found in non-accumulator plants (Assunção et al., 2003b), some of the highly expressed pathways probably contribute to these traits.

Apart from the more or less constitutive high expression of the above-mentioned pathways contributing to the basal level of tolerance and accumulation, metabolic differences can be expected between *N. caerulescens* populations adapted to environments with different metal complements. The different populations are potentially a rich source of variation in the uptake, translocation, accumulation of and tolerance to several metals, and could thus serve as useful platforms in comparative analyses to explore the underlying molecular and evolutionary mechanisms. For example, one of the most studied *N. caerulescens* accessions found near a Zn/Cd smelter in Prayon, Belgium (Vázquez et al., 1992), accumulates 3.4% Zn in shoot dry weight, with shoot to root ratio of ca. six (Monsant et al., 2011), but accumulates much less Cd than some populations from Southern France (Robinson et al., 1998; Lombi et al., 2000; Reeves et al., 2001; Roosens et al., 2003). Assunção et al. (2003c) studied the physiological differences among four *N. caerulescens* accessions: Ganges from Southern France is the best Cd accumulator; La Calamine from a calamine ore waste in Belgium enriched with Zn, Cd, and Pb has the lowest level of accumulation of all the metals tested, and accumulates much less Cd and Ni than the non-accumulator *T. arvense*; Monte Prinzera from a Ni-enriched serpentine soil in Italy is characterized by its superior Ni tolerance and accumulation but is very sensitive to Cd; Lellingen from a non-metalliferous soil in Luxembourg is relatively sensitive to all three metals. Ganges and La Calamine have similar tolerance profiles, being the most tolerant to Zn and Cd, but least tolerant to Ni. Xing et al. (2008) demonstrated large variation between ten *N. caerulescens* accessions in Zn and Cd uptake and translocation. Escarré et al. (2013) found large heterogeneity in Zn, Cd, and Ni concentrations among eighteen metallicolous, non-metallicolous and serpentine *N. caerulescens* populations in response to different cultivation conditions. These examples demonstrate highly contrasting metal tolerance and accumulation characteristics among *N. caerulescens* populations, and points to their potential for comparative RNA-Seq combined with association analyses.

Comparing just two *N. caerulescens* accessions might not allow the conclusion why one population accumulates Ni and another one Cd or Zn while sharing the same genes. For example, comparison of *N. caerulescens* accessions Prayon and Ganges suggested that *HMA4* expression does not correlate with Cd translocation efficiency (Xing et al., 2008), whereas opposite conclusion was drawn from the expression patterns of three different accessions, i.e., St-Félix-de-Pallières, Puente Basadre, and Prayon (Craciun et al., 2012), supported by our RNA-Seq comparison of Ganges, La Calamine, and Monte Prinzera accessions (Halimaa et al., 2014). However, both studies are consistent with initial findings of constitutive high expression of *HMA4* in hyperaccumulators (Bernard et al., 2004; Talke et al., 2006; van de Mortel et al., 2006; Hammond et al., 2006), which is largely due to gene duplication (Hanikenne et al., 2008; Lochlainn et al., 2011; Craciun et al., 2012). A careful combination of accessions can thus significantly improve the predictability of comparisons. It is, however, important to recognize that in RNA-Seq analysis the number of replicated samples is the most predominant factor in providing detection power for differential expression (Rapaport et al., 2013).

The application of RNA-Seq and multiple predictive tools to the comparison of several *N. caerulescens* accessions is a powerful way to provide insights into the evolution of the populations in different environments. As an example, Gene Ontology (GO) enrichment analysis indicated that the most significant differences between *N. caerulescens* accessions La Calamine, Ganges and Monte Prinzera are related to metal ion (di-, tri-valent inorganic cation) transmembrane transporter activity, iron and calcium ion binding (inorganic) anion transmembrane transporter activity, and antioxidant activity (Halimaa et al., 2014).

Co-segregation, genetic linkage mapping and quantitative trait loci (QTL) analyses make use of crosses between genotypes with contrasting phenotypes. Several crosses have been made between *N. caerulescens* accessions (Assunção et al., 2003a, 2006; Deniau et al., 2006; Richau and Schat, 2009). However, expression QTL (eQTL) has not been applied so far to address metal hyperaccumulation or tolerance. Candidate gene identification can be further improved by applying RNA-Seq to recombinant inbred lines selected for particular tolerance or accumulation capacities or, preferably, their associated QTL markers.

## PROSPECTS

Deep sequencing technologies with unparalleled accuracy, resolution and throughput have revolutionized transcriptomic and genomic research. So far full advantage of these techniques has not been taken to study the regulatory mechanisms underlying metal hyperaccumulation and tolerance. Environmental stress factors can cause changes in chromatin properties and in the production of small RNAs that contribute to regulation of gene expression. Deep sequencing has the potential to detect the small RNAs with low abundance. For example, several micro-RNAs (miRNAs) regulated by drought, cold and salt in rice, and responding to aluminum treatment in *Medicago truncatula*, were identified with deep sequencing (Barrera-Figueroa et al., 2012; Chen et al., 2012). Metals cause epigenetic changes, and epigenetics may have a role in plant adaptation to metalliferous environments, but there are only few studies of metal effect at DNA methylation level (Aina et al., 2004; Wada et al., 2004; Choi and Sano, 2007; Ou et al., 2012).

Having populations with a wide range of metal hyperaccumulation and hypertolerance capacities, *N. caerulescens* shows a great potential to serve as an excellent model in plant evolutionary genomics. Combined with high-throughput sequencing, a significant leap forward is expected in the understanding of the metabolic adaptation of this plant to different metal environments. However, even with its increasing power, RNA-Seq still remains a screening method that provides candidate genes, a more detailed analysis being needed to prove the true significance of the findings.

## Conflict of Interest Statement

The authors declare that the research was conducted in the absence of any commercial or financial relationships that could be construed as a potential conflict of interest.
